# Haboob Dust Storms and Motor Vehicle Collision-related Trauma in Phoenix, Arizona

**DOI:** 10.5811/westjem.59381

**Published:** 2023-07-17

**Authors:** Michael B Henry, Michael Mozer, Jerome J Rogich, Kyle Farrell, Jonathan W Sachs, Jordan Selzer, Vatsal Chikani, Gail Bradley, Geoff Comp

**Affiliations:** * Valleywise Health Medical Center, Creighton University Arizona Health Education Alliance, Department of Emergency Medicine, Phoenix, Arizona; † Stanford School of Medicine, Department of Emergency Medicine, Palo Alto, California; ‡ Creighton University School of Medicine - Phoenix Campus, Phoenix, Arizona; § Phoenix Children’s Hospital, Department of Pediatrics, Phoenix, Arizona; ¶ Bureau of EMS and Trauma System, Arizona Department of Health Services, Tuscon, Arizona; || George Washington University School of Medicine, Department of Emergency Medicine, Washington, D.C

## Abstract

**Background:**

The Sonoran Desert region, encompassing most of southern Arizona, has an extreme climate that is famous for dust storms known as haboobs. These storms lead to decreased visibility and potentially hazardous driving conditions. In this study we evaluate the relationship between haboob events and emergency department (ED) visits due to motor vehicle collisions (MVCs) in Phoenix, Arizona.

**Methods:**

This study is a retrospective analysis of MVC-related trauma presentations to Phoenix, AZ, hospitals before and following haboob dust storms. These events were identified from 2009–2017 primarily using Phoenix International Airport weather data. De-identified trauma data were obtained from the Arizona Department of Health Services (ADHS) Arizona State Trauma Registry (ASTR) from seven trauma centers within a 10-mile radius of the airport. We compared MVC-related trauma using six- and 24-hour windows before and following the onset of haboob events.

**Results:**

There were 31,133 MVC-related trauma encounters included from 2009–2017 and 111 haboob events meeting meteorological criteria during that period. There was a 17% decrease in MVC-related ED encounters in the six hours following haboob onset compared to before onset (235 vs 283, *P* = 0.04), with proportionally more injuries among males (*P* < 0.001) and higher mortality (*P* = 0.02). There was no difference in frequency of presentations (*P* = 0.82), demographics, or outcomes among the 24-hour pre-and post-haboob groups.

**Conclusion:**

Haboob dust storms in Phoenix, Arizona, are associated with a decrease in MVC-related injuries during the six-hour period following storm onset, likely indicating the success of public safety messaging efforts. Males made up a higher proportion of those injured during the storms, suggesting a target for future interventions. Future public-targeted weather-safety initiatives should be accompanied more closely by monitoring and evaluation efforts to assess for effectiveness.

## INTRODUCTION

Weather events are known to have a profound effect on human health. The Sonoran Desert region, encompassing most of southern Arizona, has an extreme climate that is famous for a meteorological phenomenon known as a “haboob.” The word, of Arabic origin, originally described storms in the Middle East and North Africa and was first used to describe similar events in the southwestern United States in a 1972 paper by Idso et al.^1^ The haboob is a unique dust storm occurring when desert sediment is sent airborne by the convective downdrafts of thunderstorms. The advancing walls of dust typically develop southeast of Tucson and travel northeast toward Phoenix. They can reach over a kilometer in height and are accompanied by high winds, rapid decreases in visibility and temperature, and increases in airborne particulate matter.^2^–^4^

Dust storms exert negative health effects in different ways. For example, studies in a variety of contexts suggest that particles under 10 micrometers in diameter (PM 10) from dust storms may be associated with increased hospital visits and emergency department (ED) visits, especially from cardiopulmonary disease.^5^–^11^ Locally, Dimitrova et al showed a correlation between particulate matter and childhood asthma attacks in Phoenix, while Tong et al suggest that increased dust storm activity may be linked to an uptick in fungal *Coccidioides* infections.^12^,^13^ The increase in particulate matter also leads to decreased visibility, which may lead to hazardous driving conditions.

Over five million motor vehicle collisions (MVCs) result in the deaths of around 40,000 U.S. residents every year.^14^,^15^ An estimated 16–25% of traffic fatalities may be related to adverse weather, particularly due to rain and wet conditions, costing tens of billions of dollars and thousands of lives yearly.^15^,^16^ While low-visibility conditions have been associated with MVCs and fatalities,^15^ there is limited research specifically regarding the impact of haboobs on the frequency of MVC-related trauma secondary to adverse driving conditions.

Dust storms are the third leading cause of weather-related fatalities behind extreme heat/cold and flash flooding in Arizona.^3^ Dust storm modeling has identified two dust storm hot spots for MVCs: at the intersection of Interstate 8 and Interstate 10 near Casa Grande, as well as at the intersection of I-10 and I-17 in Phoenix.^17^ According to the National Oceanic and Atmospheric Administration, 1,521 dust-related MVCs occurred in Arizona between 1955–2011 resulting in 157 deaths and 1,324 injuries.^18^

With rapid population growth in the state and evidence for climate change causing increasing severity of haboobs, understanding the effects of severe weather on driving conditions and MVC-related health outcomes is crucial.^2^–^4^ Elucidating this relationship can help improve driver safety as well as preparedness of EDs and emergency medical services (EMS) personnel. The primary outcome of this study was the change in frequency of ED visits for MVC-related trauma following onset of a haboob. Secondary outcomes assessed were changes in group characteristics of demographics, transport methods, and clinical outcome measures among those with MVC-related trauma following a haboob.

**Population Health Research Capsule**

What do we already know about this issue?

*Many motor vehicle collisions (MVC) and associated injuries are due to adverse weather conditions*.

What was the research question?

*This study explores the relationship between MVC trauma and dust storms (“haboobs”) in the Phoenix, Arizona, area*.

What was the major finding of the study?

*There was a 17% decrease (P* = *0.04) in MVC injuries during the six-hour period following haboob onset*.

How does this improve population health?

*Public safety messaging regarding adverse weather conditions may help drivers avoid dangerous conditions*.

## METHODS

This was a retrospective analysis of MVC-related trauma presentations to Phoenix, Arizona, hospitals before and after haboob dust storm events from 2009–2017. This study was approved by the Arizona Department of Health Services (ADHS) Human Subject Review Board and the Valleywise Health Institutional Review Board.

### Identification of Haboob Events

Haboob events are characterized by a unique array of meteorological patterns that are relatively rapid and intense compared to other types of storms. Most distinguishing is the significant decrease in visibility, along with an acute rise in the amount of dust and high winds, and a rapid drop in temperature. These changes are observed from the baseline meteorology over a period of hours. There is no established strict meteorological definition of haboob, nor is there a quantifiable method of distinguishing one from other dust storms. For the purposes of this study, we developed a set of criteria to define haboob events based on previously published attempts at identification and simulation of haboobs in the region.^1^,^4^,^18^

Haboob event criteria consist of an acute drop in visibility to less than or equal to seven miles at Phoenix Sky Harbor International Airport weather station (KPHX) within two hours of either

1. An acute rise in PM10 to greater than or equal to 200 μg/m^3^ in diameter, or
2. An acute rise in PM10 to greater than or equal to 100 μg/m^3^ and wind gusts greater than 38 miles per hour.

Haboob events were identified from publicly available historical climate and air quality data for the period of January 1, 2009–December 31, 2017. Weather data for visibility and wind were obtained from the National Climate Data Center as a Local Climatological Dataset sourced from the KPHX weather station.^19^ These data represent the point conditions at KPHX in central Phoenix, the most comprehensive and widely referenced weather station in the metropolitan area. Particulate matter datasets were requested from the Environmental Protection Agency Air Quality System, and daily maximum PM10 was obtained from the nearby air monitoring sites at Central Phoenix (site 04-013-3002), Tempe (site 04-013-4005,) and South Scottsdale (site 04-013-3003,)^20^ Of note, the Tempe and South Scottsdale air monitoring sites did not go online until 2012. One of the authors (JW Sachs) requested and processed data from the above sources. Particulate and weather data were combined and cleaned in Excel 2019 (Microsoft Corporation, Redmond, WA) and visualized using Power BI 2019 (Microsoft) and TimeSeries 2019 (ZoomCharts, Riga, Latvia).

The identification process involved the manual review of plotted climatological data. While haboobs are known to be associated with rapid drops in temperature, the utility of this variable was poor. Classification was made challenging due to quality problems with the climate and air quality datasets. For example, daily PM10 and wind gust values were frequently missing. When severe inconsistencies arose, we attempted to correlate a suspected event with an online eyewitness account or archived news report.

### Identification of Motor Vehicle Collision-related Trauma

De-identified trauma data were obtained from the ADHS Arizona State Trauma Registry (ASTR) from 2009–2017. Included patient encounters were all involved in MVC-related trauma as defined by “Data Element I2_14a: CDC Mechanism/ Cause Category for Primary ICD-10 External Cause Code.” All ages were included. Data fields collected included demographics (age, gender, state, and country of residence), accident details (date and time of injury, ZIP code and county of injury),method of transportation, and outcome measures (ED disposition, total length of ED plus inpatient stay, total hospital charges, and mortality outcome).

Literature on the size of haboobs in Arizona is limited and widely variable, with storm sizes 10–20 miles in length described at Williams Air Force Base ranging up to witnessed storm walls over 100 miles long outside Phoenix.^1^,^18^,^21^ To include encounters from areas most likely to be affected by haboob conditions, presentations were included from the seven Level I-designated trauma centers within 10 linear miles of the KPHX weather station. Injuries that occurred outside Maricopa County were excluded as these MVCs were deemed less likely to be linked to the haboob event measured at KPHX. Also excluded were records where the time and date of injury were not explicitly recorded.

### Data Analysis

Data analysis was conducted using JASP version 0.14.1 (Jeffrey’s Amazing Statistics Program, University of Amsterdam, The Netherlands, 2021). Basic descriptive statistics were performed on the entire dataset of all MVC-related encounters from 2009–2017.

As with data regarding the size of haboobs, data on their duration in Arizona is limited and variable, and some commonly cited sources use data from other countries.^22^,^23^ Brazel described dust-related visibility reductions in Arizona occurring 78 minutes prior to and 229 minutes (SD 220) after the storm front, while Idso reported an average of one hour but up to three hours.^1^,^24^ Eagar found that high particulates are found up to one to two hours during haboobs, while some milder dust storms had conditions lasting up to 12 hours.^4^ Crooks reported that while half of haboobs lasted less than one hour, a significant number lasted longer than seven hours.^11^

Based on these studies, to create an inclusive time window capturing the majority of MVC injuries potentially linked to a haboob event we defined “post-haboob” MVC trauma as occurring up to six hours after the onset of haboob conditions. The control group of “pre-haboob” MVC trauma includes injuries occurring in the six hours leading up to haboob onset. Additionally, we repeated the analysis using a 24-hour window before and after haboob onset to assess whether lingering weather conditions were linked to changes in MVC frequency. The frequency of MVC-related trauma in the pre-haboob (control) and post-haboob (exposure) groups were compared using binomial analysis. Additionally, pre- and post-haboob MVC trauma was compared with subgroup analyses for demographics, transport methods, and outcome measures using the Student *t*-test for continuous variables and chi-squared test for categorical variables.

Occasionally, several haboobs occurred in rapid succession, for example, with a patient simultaneously qualifying as “post-” (the earlier haboob) and “pre-” (the subsequent haboob). In these cases, we considered the patient to be in the “post-haboob” group of the first event and discarded subsequent encounters linked to later overlapping haboobs.

## RESULTS

From January 1, 2009–December 31, 2017, there were 31,133 patient encounters meeting criteria (MVC-related trauma presenting to one of the seven included hospitals within 10 miles of KPHX).The mean age of all encounters was 35.8 years (SD 19.6, range 0–101), and 58.9% of the patients were male. The ED disposition of 67.3% of the patients was admission from the ED; 29.6% were discharged, 2.0% died in the ED, 0.6% required transfer to another hospital, and 0.4% bypassed the ED as a direct admission. Arizona state residents included 97.0% of the patients. Ground ambulance transported 88.5% and 6.7% via helicopter, while 4.5% were ED walk-ins. The mean total length of stay for all patients (ED plus inpatient) was 3.3 days (SD 6.7 days, range 0–309 days). The mortality rate was 4.1%.

### Haboob Events

**Figure 1:**
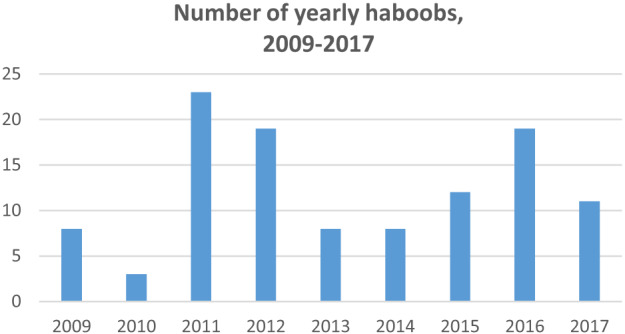
Number of haboobs per year from 2009–2017.

**Figure 2:**
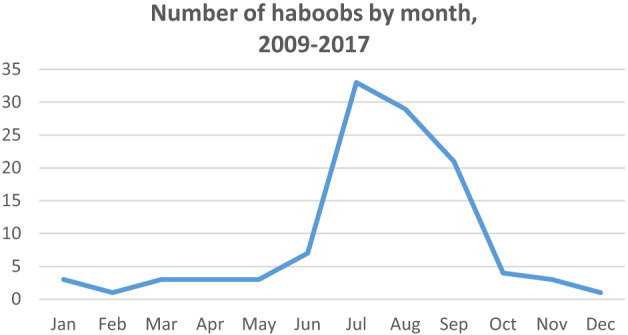
Number of haboobs by month, averaged from 2009–2017.

From 2009–2017, there were 111 haboob events meeting our inclusion criteria. The maximum number of haboobs in a single year was 23 (in 2011) and the minimum was three (in 2010), with a mean of 12 haboobs per year (SD 6.6). The greatest number of haboobs during these years happened in the months of July (33), followed by August and September, respectively (29 and 21). See Figures 1 and 2.

### Haboob-related MVC Patient Presentations

Analysis using 24-hour pre- and post-haboob periods revealed that 815 patients were injured pre-haboob compared to 825 in a post-haboob period (*P* = 0.82). There was no demographic difference between these groups regarding age, gender, or Arizona residence (*P* = 0.10, 0.21, and 0.53, respectively). A similar number of patients in the 24-hour pre-and post-haboob periods were transported by helicopter compared to ground ambulance in both groups (*P* = 0.07). Outcomes of total length of stay, hospital charges, and mortality were also similar in both groups (*P* = 0.25, 0.88, and 0.80, respectively).We excluded 143 patient encounters (8.0% of MVC injuries within 24 hours of a haboob onset) due to overlapping haboob timeframes.

Analysis using six-hour pre- and post-haboob periods demonstrated a roughly 17% decrease in MVC injuries in the six-hour period following haboob onset (235 vs 283, *P* = 0.04). The six-hour post-haboob period had a proportionally higher number of injured males (*P* < 0.001), while there was no difference in age or Arizona residence (*P* = 0.23 and 0.50, respectively). No significant difference was noted in helicopter vs ground ambulance transport (*P* = 0.06). While LOS and total hospital charges were similar in six-hour pre- and post-haboob patients (*P* = 0.52 and 0.82), there was a higher mortality noted in the post-haboob period (*P* = 0.02). Four patient encounters (0.008% of those within six hours of a haboob onset) were excluded from two occasions of overlapping haboob timeframes. Please see Table 1.

## DISCUSSION

This study evaluated the relationship between haboob dust storm events and MVC-related ED visits in Phoenix, AZ. Prior studies have demonstrated the general relationship between bad weather and poor visibility and MVC fatalities,^15^,^16^ and quantified haboob-related MVCs and associated morbidity and mortality in Arizona.^3^,^17^ However, we are not aware of any study comparing the number of MVC-related injuries following haboob onset compared with baseline, or any study describing changes in the numbers of MVC-related injury cases presenting to local EDs around the time of a haboob event.

Interestingly, the frequency of MVC-related injuries decreased during the six-hour period following haboob onset by approximately 17%. No significant decrease was seen when considering presentations during a 24-hour period following haboob onset; this may be because the duration of most haboobs is only several hours 1,4,11,24 and, therefore, a 24-hour period includes many non-haboob-related incidents, with the misclassification biasing our results toward the null. This drop in injury frequency noted during the six-hour post-haboob period may be due to changes in driver behavior secondary to public safety messaging systems. A large haboob-linked multicar collision in 2011 led to collaborations between the Arizona Department of Transportation, the National Weather Service, Arizona Department of Public Safety, and the Governor’s Office of Highway Safety in rolling out the “Pull Aside, Stay Alive” campaign. The campaign has included radio alerts, television messaging, and increased roadside signage, even encouraging public participation via whimsical aspects such as the “Haboob Haiku Challenge.”^25^

Current robust, public health messaging in Arizona may be successful in keeping drivers off the road and safe during haboobs, similarly to what was noted with a decrease in MVCs in Virginia following a rollout of wireless alerts regarding flash floods^26^ and in southern California with the use of changeable message signs.^27^ A Canadian study in 2010 similarly showed that risk of MVCs associated with rainfall significantly decreased over two decades, which could partially be attributed to driver behaviors, in addition to road and vehicular improvements.^28^ Although MVC injuries decreased during the six hours following haboob onset, there was a higher proportion of fatalities among cases presenting to the included EDs, suggesting that those who did not stay off the roads were involved in more serious collisions; however, other outcome metrics of length of stay and hospital charges did not suggest a difference.

While males were overall more likely to suffer from an MVC injury, consistent with prior trends secondary to risky behaviors and number of miles driven,^29^ males were also disproportionately represented among injuries occurring during the six-hour window following haboobs. One study found that females may be superior to males at keeping within their lanes during foggy weather conditions,^30^ and even in non-US contexts surveys suggest that male driving behaviors may exacerbate risks secondary to inclement weather.^31^

It was hypothesized that non-Arizona residents may have represented a larger number of MVCs during haboobs due to unfamiliarity with these weather events, but there was no difference in residency among those injured in haboobs. There was an apparent difference in both the six- and 24-hour groups, although ultimately statistically insignificant, in transport mode with proportionally fewer helicopter EMS following haboobs, unsurprising given the association with bad weather and an increased number of fatal crashes.

## LIMITATIONS

This study had several limitations. While we conducted the analysis under the presumption that MVC injuries occurring in the hours following haboob onset could be linked to weather, the datasets we used did not explicitly say whether the weather was a causative factor in the MVCs. Weather data was not available on the exact spatiotemporal patterns of the storms, and trauma data included ZIP codes as opposed to specific intersections; thus, beyond including cases presenting to hospitals within the radius and excluding injuries sustained outside Maricopa County, there was no more clear way to more precisely link weather and the MVCs. While we attributed changes in MVC frequency following haboob onset to weather conditions and visibility, there are confounders that could not be assessed in this study (such as substance or cell phone use or vehicle mechanical issues), but were likely controlled in our analysis. Additionally, because pre-/post-classification of MVCs occurring during periods when multiple haboobs happened in rapid succession was impossible, these events were removed. They represented only a small proportion of encounters, and their removal did not bias the results.

Selection of the controls for comparison to post-haboob injuries was challenging, since many changing traffic conditions could affect MVC and injury frequency (for example, decreased MVC numbers following evening storms may have been more related to natural fluctuations in commuter numbers). However, we decided that despite time-of-day variations in traffic, the hours preceding haboobs events were otherwise the best controls, and with enough included haboob events the daily traffic variations between pre- and post-haboob time periods were negligible.

Another limitation was due to dataset completion; for example, while many fields were consistently filled out on the ASTR, others like total hospital charges were not available for many cases. Additional information for injury characterization was also unavailable; therefore, mortality, length of stay, and hospital charges were used as proxies for injury severity. The available data likely underestimated trauma because not all MVC-related injuries led to activation of hospital trauma teams (and there was likely variation in trauma team activation between institutions unaccounted for here); thus, there may have been a number of minor injuries not included, while incidents of MVC-related mortalities that were not transported from the scene also would not have been included.

## CONCLUSION

Haboob dust storms in Phoenix, Arizona, may be followed by a decrease in motor vehicle collision-related injuries during the six-hour period following storm onset, perhaps due to the success of public safety messaging efforts. Males made up a higher proportion of those injured during the storms, suggesting a target for future interventions. Future public-targeted weather safety initiatives should be accompanied more closely by monitoring and evaluation efforts to assess for effectiveness.

## Figures and Tables

**Table 1:** Demographics and outcomes of patients presenting within 24- and six-hour windows following haboob event onset.

	All	24-hour haboob period			6-hour haboob period		
	
		pre-	post-	*P*-value	pre-	post-	*P*-value
MVC injuries (# patients)	31,133	815	825	0.82	283	235	***0.04***
Age (mean years)	35.8	34.6	36.2	0.10	33.8	36.0	0.23
Gender (% male)	58.9	58.2	61.2	0.21	51.2	65.5	<***0.001***
Place of residence (% Arizona)	96.4	96.9	96.4	0.5	96.1	94.8	0.50
Transport (% heli vs ground ambo)	7.0	7.4	5.1	0.07	7.0	3.2	0.06
Total ED + admit LOS (days)	3.3	3.5	3.1	0.25	3.5	3.0	0.52
Total hospital charges (mean $)	65,760	63,913	63,104	0.88	60,631	63,056	0.82
Final outcome (% mortality)	4.1	2.8	3.0	0.80	1.1	4.3	***0.02***
Excluded (# patients)		143			4		

*MVC*, motor vehicle collision; *ED*, emergency department; *LOS*, length of stay; *heli*, helicopter; *ambo*, ambulance.
